# Supplementary material for: Merit and placement in the American faculty hierarchy: Cumulative advantage in archaeology
Source: PLoS One. 2022 Jan 31;17(1):e0259038. doi: 10.1371/journal.pone.0259038 (PMC8803199; doi:10.1371/journal.pone.0259038)
Supplement: S1 File — (DOCX) [file pone.0259038.s001.docx]

Supporting Information 2.

Later review of data files identified minor discrepancies in *h* as calculated from PoP’s Google Scholar database in 11 of 131 cases (Table S2). “Difference” reflects direction and magnitude of error in “h Original.” Most discrepancies are slight; six of 11 original values exceed revised *h* by one, and absolute value of discrepancies is 1 in eight of 11 cases. The average of these discrepancies, and of absolute value of discrepancies, is slight. Revision occurred to original search results, not a second, later search that might update results with additional citations.

I attempted no revision in any other bibliometric measure, which are much more tedious to calculate by hand than is *h*. Such revisions would best be accomplished by new searches months after original data collection, which itself would require similar updates to all 131 case, 120 of which had no discrepancy. Discrepancies were noted after analysis was completed. They are few in number and small in magnitude, so otherwise ignored here.

Table S2.

| NAME | h Original | h Revised | Difference |
| --- | --- | --- | --- |
|  | 15 | 13 | 2 |
|  | 2 | 3 | -1 |
|  | 4 | 3 | 1 |
|  | 31 | 27 | 4 |
|  | 4 | 3 | 1 |
|  | 26 | 25 | 1 |
|  | 8 | 6 | 2 |
|  | 19 | 18 | 1 |
|  | 11 | 10 | 1 |
|  | 29 | 30 | -1 |
|  | 17 | 16 | 1 |
