# Supplementary material for: Merit and placement in the American faculty hierarchy: Cumulative advantage in archaeology
Source: PLoS One. 2022 Jan 31;17(1):e0259038. doi: 10.1371/journal.pone.0259038 (PMC8803199; doi:10.1371/journal.pone.0259038)
Supplement: S2 File — (DOCX) [file pone.0259038.s002.docx]

SUPPORTING INFORMATION 3

In archaeology and its cognate field of anthropology, one major journal—Current Anthropology—contains a feature that complicates the calculation of bibliographic indices. Each issue of Current Anthropology includes several “comment” articles. The text of each such article is followed by short comments—roughly 1,000 words each—from as many as 10 scholars with expertise on the article’s subject. GS records each of those scholars as a co-author of the paper.

This practice does not directly inflate publication counts, because those brief comments are in fact published. It does, however, credit each commenter with a paper in Current Anthropology when he or she only drafted short comments in response to an original contribution. What is more, indices that seek to correct for multiple authorship by parsing credit equally among co-authors mistakenly awards equal credit to the original author and all commenters. That treatment favors commenters in one respect, because it artificially inflates their citation numbers and, possibly, their bibliometric values. Yet it also can disadvantage them, because parsing for co-authorship artificially inflates commenters’ co-author counts. In my own case, for instance, PoP via GS reads *h*-core publications as including many more co-authors than I actually have collaborated with, thereby artificially deflating “corrected” index value. No less is true of other archaeologists who have authored major papers in Current Anthropology and/or have commented on others’ papers.

Over 20 archaeologists in the sample had published either original papers or comments in Current Anthropology, some of them more than once. Sixteen of these scholars had published comments there. I made no adjustment to *h* for archaeologists who authored or co-authored original papers in that journal, reasoning that they had attracted most or all of the citations, nor any attempt to correct *hIann* for exaggerated co-author counts. For comments contributed by indexed scholars, I corrected search results by deleting the entry, which removed the unearned citations from the scholar’s record, then manually adding one to his or her publication count—but not citations—to restore the comment entry that the deletion removed.
