# Supplementary material for: Merit and placement in the American faculty hierarchy: Cumulative advantage in archaeology
Source: PLoS One. 2022 Jan 31;17(1):e0259038. doi: 10.1371/journal.pone.0259038 (PMC8803199; doi:10.1371/journal.pone.0259038)
Supplement: Attachment — Submitted filename: Response to reviewers 2.docx [file pone.0259038.s004.docx]

# Department of Anthropology and Classical Studies

Buchtel College of Arts and Sciences

Akron, OH. 44325-1910

(330) 972-7875 Office

28 Sept. 2018

Dr. Cassidy Sugimoto

[sugimoto@indiana.edu](mailto:sugimoto@indiana.edu)

re: revised ms., PONE-D-17-44425R1 “Merit and Placement of Archaeologists in the American University Hierarchy”

Dear Dr. Sugimoto:

This is my second “response letter” about this ms. References to line numbers pertain to the revised version, PONE-D-17-44425R1,except where the re-revised ms. is indicated.

OK, now we have two additional reviews, one of which (#5) is strongly positive. That reviewer has a few minor reservations (I too have concerns about any ranking, as the original and revised version [lines 259-64] stated). On several of those minor points:

1. Line 120-21’s personal interactions occurred in the past so were not observed whether or not they were observable but, yes, can be observed if we have observers at interview lectures, personal meetings, and dinner conversations.
2. Degree program as “not…an important student criterion”. Inserting “undergraduate” before “student” should clarify, or the reference to students can be omitted from the sentence. By this (minor) statement I meant that undergrads probably don’t care which or how many graduate programs their undergraduate school offers. Small point, addressed by a small change in the text.
3. Omitted “s” on line 220 noted, as is “than” for “that” on line 641 and the extra period on 1065.
4. Finally, if necessary I can cite the sociology source identified on criticism of NRC Sociology rankings.

By now, then, we have three strongly positive reviews, plus #1 and #6. I’ve already expressed my opinion of #1, supported by detailed empirical and logical argument. That review is irredeemably biased and, despite its tedious length, almost entirely unsubstantive. If you continue to take it seriously, I am entitled to know how and why my detailed refutation of its various unsubstantiated claims failed to address those claims. That leaves #6.

Reviewer 6. Much of this review is devoted to a detailed discussion of…the abstract, which it practically parses, laboring mightily to quibble and contort. That makes me wonder, obviously, how much of the paper #6 actually read. Because s/he noted typos there, I know that #6 read at least lines 1065 and 1100. By the way, near both start and end #6 states his/her agreement with “the other reviewers” when in fact by now three of five have delivered strongly positive, slightly qualified reviews, none of which made remotely “extensive” comments about my prose. This comment strikes me as a cooperative response to editorial coaching, if not directly (e.g., “Please tell him that his writing is poor…”) then by strategic choice of reviewer. (It’s clear from your own writings, for instance, that you work with Gingras and Lariviere. Whatever views you may share with them, as an editor of course you have the obligation to treat mss. fairly and without regard to your own views.) No reviewer—not even #1—gave “extensive discussion” to writing style or clarity, although #1 did impugn it without substantiating the charge, this in a review text that included such sparkling gems of English prose as “prioritization” and “self-indulgery.” I’m told that I write poorly by people who don’t write as well, always a treat to experience, and in the form of blanket charges that lack evidence or examples to support them.

Except, of course, by #6 on the abstract, which isn’t the place to discourse at length on definitions or to make in detail the case that the *abstract* cannot make. Merriam’s defines “abstract” as a “[summary](https://www.merriam-webster.com/dictionary/summary#h2) of points (as of a writing) usually presented in [skeletal](https://www.merriam-webster.com/dictionary/skeletal) form” (original emphasis). *Skeletal* form. Yet #6 criticizes my abstract for failing to flesh out my argument in all details. Summaries—abstracts—by their nature cannot and should not attempt to present arguments in nearly all relevant detail. On the contrary, good ones should state the problem or question, describe data compiled and analysis conducted to address the question, and state conclusions. All briefly. More accurately than s/he her/himself puts it, then, #6 agrees with *one,* not all reviewers, although #6 is slightly less negative than is #1.

I’m sorry, but I can only discount comments about my writing the quality of these, from a native English speaker or anyone else. The use of “pattern” as a verb is well-established in academic English and requires no justification. (While I’m consulting Merriam’s, let’s see if it treats “pattern” as a verb. What a surprise: it does! And Merriam’s defines it exactly as I used it. So let’s dispense with that illegitimate criticism.) The review of the abstract offers repeated cricitisms about terms undefined there, all of which are defined in the text. Should abstracts now be devoted to definition of terms? Why not include citations? Section headings? Perhaps footnotes? Tables and figures? Abstracts are to summarize, not to document in detail, at least as Merriam’s and I understand it. #6 is entitled to disagree with me, as you are. But let’s also consult PLoS One on the matter. Here is how the journal, which you serve as an editor, describes abstracts: “The Abstract should:

- Describe the main objective(s) of the study
- Explain how the study was done…without methodological detail
- Summarize the most important results and their significance
- Not exceed 300 words”

Seems reasonable to me, and consistent with my practice over the past 35 years. Yet #6 criticizes my abstract for failing to describe what, in this context, can only be called “methodological detail” and not merely summarizing, but documenting in detail, its “most important results and their significance”. In effect, #6 criticizes the abstract for conforming to PLoS One editorial standards. Who is s/he arguing with? To what argument or requirement am I expected to reply? To my eye, this review’s comments about writing style and clarity of expression strain to differ and, again, read as though they were coached.

On #6’s specific comments about my abstract:

- I fail to make clear “the distinction between MA and PhD-granting programs.” Ms. lines 198-200, among others, cover this point. #6 then accuses me of poor writing because the distinction’s salience “only becomes clear later in the paper…” Yes, *in the paper.* Not in the abstract. This is an absurd criticism: I fail to make my full argument in an abstract that PLoS One itself tells me is not to be used for that purpose.
- The abstract’s third sentence fails to define “ranked subsets of PhD programs.” This criticism is so urgent that #6 states it twice. But of course I didn’t define those subsets there; the abstract isn’t the place for the definition. Had #6 troubled him/herself to read, say, ms. lines 927-978, a section that treated ranked subsets of PhD programs, s/he would have found the definitions on lines 935-36 and 955-56.
- The next comment demands insertion of a comma two words after an existing comma. I’ve reworded, slightly and differently, to clarify what I honestly believe most readers would understand clearly.
- The parsing continues by professing bewilderment at what “mobility” may mean. Again, recalling that PLoS One urges brevity and summary, not detailed disquisition, I can only refer #6 to the actual text, which the reviewer should have read carefully before presuming to criticize the abstract. Had #6 bothered to read it, s/he would have encountered the definition on ms. lines 834-40, especially 839-40. It all seemed reasonably clear to me, but re-revision rewords further. At the same time, I welcome any specific, constructive suggestion to make the passage even more clear.
- #6 writes “One can only guess” about a supposedly unclear point, also related to mobility. One also could read the text, not just the abstract, again in this case lines 838-40.
- The abstract’s sixth sentence—golly, #6 certainly does enjoy enumerating abstract sentences, presumably to make his/her point, and cleverly so, about how the abstract reveals at once my flaws as writer and scholar—states a conclusion that #6 doesn’t like. But #6 doesn’t admit as much, instead suggesting that the difference in averages by program level is inconsistent with the conclusion. That is a distortion of my argument and, for that matter, Long’s and Merton’s before me. It focuses exclusively upon career measures, and deliberately avoids their inconsistent patterning with precocity ones which, of course, is a key component of the analysis. The entire criticism reduces to a dishonest, half-clever ruse intended to misrepresent rather than engage with the analysis.
- #6 then concludes that “the abstract already took me a good deal of time to understand correctly.” Two things. First, #6 does *not* understand the abstract correctly as indicated by the plain reference to text passages that explain what s/he condemned the abstract for failing to describe or document in detail (i.e., criticizing the abstract for what it cannot be). Nor does s/he take it at face value, as indicated by the deliberate distortion of my logic and conclusions noted above. None of #6’s failings here owe to any expository flaws of the abstract or text, merely to her/his absurd treatment of abstracts as complete arguments. Second, #6 either is obtuse, in which case I can only question her/his competence to review the ms., or disingenuous. But surely, it “already took me a good deal” *more* time tediously to refute his/her unsubstantiated criticisms and respond to his/her charges that the abstract must do what only the text may do. #6 is free to waste all of his/her time that s/he pleases, but I resent the necessity of wasting mine to respond to his/her unprofessional, unfair criticism.

Yours to me of 20 August expressed “concerns about the readability of the manuscript,” which I take as an implicit requirement to improve that “readability.” Apart from minor comments that three reviewers made about wording, to which I’ve responded, the “concerns” were expressed by #1, #6 and you. As above, #1’s criticisms were severe; recall that s/he rated the paper “incoherent,” presumably #1’s prioritized hot take. Alas, those criticisms weren’t merely not *well*-documented; they weren’t documented at all. My earlier response dealt directly with #1’s broad, ill-founded, objections. Now, #6. I discount #6’s tedious parsing of the abstract as a clumsy attempt to criticize my writing by failing—probably deliberately--to understand the purpose of abstracts. I will not change words like “pattern” to please a single nonnative speaker who considers it unusual. Any substitute might “confuse” other nonnative speakers who evidently don’t comprehend standard English as well as they believe they do, and anyone who presumes to read journals that publish in standard English reasonably should expect to command that language. I do not consider myself an exceptional writer, yet compared to the standards of my field—and also yours, many papers in which I subjected myself to in the past 18 months—somewhat above average. Criticisms so broad, vague and undocumented are no basis for “improving readability” of a perfectly readable ms. They are merely cheap accusations. There are no significant, legitimate “concerns” to which I can respond.

Yet your judgment is that I must respond to the accusa—er, sorry, “concerns.” So you must decide: will #1’s and #6’s unsubstantiated charges (and, in #6’s case, at once tedious and frivolous abstract-parsing) stand against three other reviews that made minor, passing editorial comments and against my detailed, substantive responses to the “readability” charge? If so, then I consider myself entitled to better documentation of my prose flaws than the risible parsing of an abstract that presumes that abstracts must present the totality of an argument, including definition of terms. #1 and #6 were incapable of documenting the broad charges they made. Are you capable? If your answer is “no,” then please save yourself the trouble of reading further. The ms. is irredeemably “unreadable.” There is no need to consider the rest of my response, to what at face value appear to be legitimate, substantive comments.

Otherwise, on to #6’s sometimes substantive comments. #6 does not repeat #1’s error about precocity measures. Those measures do not necessarily “reduce” to number of publications (P), but differences are apt to be slight for young scholars. That’s fair enough. #6 then states that there is a “clear correlation” between measures and program level in career measures, also correct. The correlation is in fact statistically significant although #6 might have noted, as the ms. does (lines 785-790), the considerable overlap in measures between program levels, and between NRC sub-groups. Even the abstract made that point (lines 43-5). Yes, there’s a pattern. But there’s sufficient diffusion in it to refute the argument—implicit in #6’s comments—that there is strong correspondence.

Now let’s turn to #6’s “three objections” to the ms.’s qualified conclusion.

His/her Point 1.

Like #1, #6 reduces merit--I presume s/he means precocity merit—to P. Let’s be as clear here as I feel an unbiased reader of the ms. would be. In both precocity interval and over the entire career, *all* analysis is conducted using cited bibliometric measures, not P. (Table 1 lists mean publications by category, for comparison.) So no, merit is not “now” or at any other time defined as P. This is exasperating. The study design requires productivity measures that pertain to the period of initial hire. If #6—or #1 previously—had constructive suggestions about other, presumably better, such measures or for study redesign, I’d give them serious regard. Instead, they merely point out the same limitation of those measures *that the ms. itself* (lines 606-07) *freely acknowledged,* as though it were both an original observation and a damning flaw of the study. Not constructive.

First, I must repeat what ms. lines 612-17 already stated, and whose purpose and point I would have thought were clear to a fair reader. Precocity measures correlate strongly with one another, and career measures correlate strongly with one another. However, precocity h correlates at much lower r (and corresponding r^2^) values with career h, as do other precocity measures with their career equivalents. That is, precocity measures provide comparatively little basis for predicting career performance. The re-revised ms. underscores this point. Reluctantly, I also attach text and figures that could be added to the re-revised ms. after line 583, although I hope it wouldn’t be necessary.

#6 then freely speculates in support of his/her universal ideal, in ways strikingly reminiscent of the Coles’ views summarized in ms. lines 107-124, and 149-150. Note line 112’s “’unproven assumption’”; these are not my words but the Coles’ own. The best account they could give of their objection to any suggestion of particularism was an assumption! I belabor this small point only because #6 invokes similar assumptions. S/he considers it “very reasonable to suppose” these shrewd judgments that bibliometric measures cannot reveal (of course not) but that must have occurred. On the strength of logic of such quality, #6 declares my conclusion a “far stretch.” It is no farther, a good deal shorter, stretch than to invent “reasonable” suppositions to the contrary. At least I have a considerable body of evidence to support my view. As editor, you might think that the ms. must be held to a higher standard than #6’s free conjecture. That’s half-right. Reviews have no obligation to make an ms.’s case for it, but in order to be taken seriously their criticisms must be based on something better than supposing, without a shred of supporting evidence, that “quality, creativity or novelty” (however novelty etc. can be gauged in any way that is not inherently particularist) win out and therefore refute a considerable body of evidence that supports a reasoned conclusion. That’s special pleading, not serious argument or fair criticism.

Ms. lines 117-124 (and 160-61) anticipated #6’s critical ploy, so essentially I’ve already replied to this objection. Please read them carefully. Then please tell me what argument of any kind or quality cannot be disputed by “reasonable suppositions” to the contrary? #6 is entitled to dispute, but to be considered fair and taken seriously the dispute must be grounded, not a matter of free supposition. Special pleading is an all-purpose bludgeon, against which there is no defense. That isn’t because it is valid, but precisely because it cannot itself be disputed on either empirical or logical grounds. Yes, it’s always possible that there may be some other factor to explain a pattern, about which free supposition can freely suppose. Only fair criticisms deserve to be respected, and they do not rely upon such poor arguments. If, if, maybe, maybe.

It’s a small point in this context but, with respect to whatever #6’s field is, “geography or methodology” are in fact highly relevant hiring criteria in archaeology. If you or #6 disagree, I invite you to read the last 30 years’ position announcements in archaeology. #6 also might have noted ms. line 1089, which cited an earlier study I wrote that clearly demonstrated exactly the pattern in geographic preference that s/he dismisses. I don’t criticize #6 for not knowing about archaeology’s clear geographic biases of long standing, but am astonished at the presumption that s/he knows the field and that such bias does not exist. This comment is fatuous nonsense.

His/her Point 2.

I’m uncertain what is meant, but intrigued by one possibility. #6 “reconsiders” the documented absence of correlation between productivity and placement in the precocity interval (note that s/he takes at face value the equally documented correlation in later career) because the ms. “effectively selects on the longevity of the career.” By this #6 evidently suggests a difference in career length between program levels that is hidden or overlooked in the study and that explains the pattern in precocity values. The evidence to support such a charge? Supposition. Thus, #6 supposes that some would have left academia; in a population of this size, about 875, that’s likely. Fair enough, again. How those departures are distributed in time or by program level is a different matter. To that degree, #6’s argument is fair if one: 1) *supposes* that those who left had low precocity scores; 2) *supposes* that they were hired by no-account universities like mine, and; 3) *supposes* that they left because they failed to meet even the presumably low standards of no-accounts like mine. *If* all of these suppositions hold then yes, there is bias “inherit to the design of the study”. (Knowing the challenge of writing in a nonnative language, I don’t ordinarily criticize the English prose of nonnative speakers. But #6 presumes to write English well enough to lecture me on how to write.) Again, this is supposition, in this case more involved, even tortured, but at least more seriously reasoned that waving the magic refutation wand of “supposing” that novelty, etc., are rewarded at hire. I can’t disprove it any more than I—or even #6, come to think of it--can prove a negative.

But consider that *if* #6’s scenario holds, then there should be significant difference in faculty proportions by professorial rank. If low-quality losers are being hired (unwisely) by no-accounts who later experience a form of buyer’s remorse by (presumably wisely, if ruefully) refusing them tenure, then faculty in lower program levels are being replaced more often. Therefore, that part of my sample should be skewed toward lower professorial rank, higher program levels toward higher professorial ranks. No such pattern is found in my data, as ms. lines 704-15 already reported. On the contrary, proportions of professorial rank by program level are similar. I have evidence, not supposition, to support this conclusion. Thus, the study does *not,* as #6 charges, “select upon the longevity of career,” because career length does not differ by program level or NRC subsample. The re-revised ms. makes that point against #6’s supposition. Actually, against #6 I might—what’s the word?—*suppose* that the process s/he describes might play out more often in PhD programs. If so, then those, not lower, programs should skew toward younger faculty and lower professorial ranks. Again, however, there is no such pattern in the evidence. Reluctantly, I am forced to add a new section (re-revised ms. lines 794-819) merely to refute this dubious argument. I confess that the logic it sets forth is tortured, but that is only because #6’s free supposition fits that description.

Finally on this point, #6 again sees ambiguity, this time in what I mean by “mobility.” This point merely repeats his/her criticism of the abstract, addressed above.

Point 3.

Sigh. Point 3 reduces to #6’s Point 1. The magic wand reappears, and free supposition freely reigns. I do not necessarily agree that capabilities are any more poorly measured in the precocity interval than the later career one. I agree that those talents are measured “by long-term productivity” *only if* controlling for the effects not just of time but the setting—here, program level—in which that time is spent. If by such ambiguous logic #6 means that savvy hiring committees see the *je ne sais quoi* that precocity measures fail to measure but that career measures can (again, #6 is quite happy to accept, without reflection or examination, a correlation b/w career measures and program level that supports his/her a priori view, but labors mightly to suppose this or that against a mucher lesser of correlation that opposes it), then I must request an explanation. How is it that career measures capture that ineffable essence while precocity measures do not? You can’t have it both ways. If you like bibliometric measures over the entire career, you can’t dismiss those that are compiled over the precocity interval, nor how they pattern—there’s that verb again—relative to career measures, merely because they are inconvenient to your preconceptions.

Lines 113-24 address objections the nature of #6’s, and before s/he even expressed them. I’m disappointed that I must call to #6’s—and your—attention something already stated in the ms., but evidently it’s necessary: “Invoking such unobservable factors against a particularist

conclusion at once renders it difficult to prove and its universalist alternative difficult to

refute.”

Let’s trace out #6’s “logic,” if that word applies in this case. If the evidence supports universalism, then the universalist conclusion must be reached. If the evidence opposes it, then the universalist conclusion *still* must be reached, in this case by free supposition that invents convenient causes. This is not merely transparently unfair but a tautology as well. However the evidence patterns—there’s that, er, verb used as a verb—or can be distorted by free supposition, it supports #6’s predetermined conclusion.

In #6’s mind, what evidence is necessary to support a qualified conclusion of particularism? Perhaps I should have attended the search interviews of all candidates—not just the hired one—of all positions at all universities in my sample over all of the past 30% years. Then perhaps I could have collected the evidence of “novelty” etc. that may *not* have set apart the favored candidate from the rest, even though s/he was hired anyway? Would that suffice? How about if I eavesdropped on the many anthropology departments during hiring-committee meetings and recorded members saying things like “Candidate x doesn’t stand out on scholarship, but we’re in the same social club so I think we should hire him?” or sentiments to that effect. Would that suffice? Presumably either example would, but such standards are absurd. I’d love to see the criminal-justice system try to operate if the only acceptable evidence of guilt were clear videos of crimes in the act of commission, the criminals being careful to face the camera for long front- and side-view poses, along with 100 sober nuns as reliable eyewitnesses. Such “standards” return us to the Coles’ “’real-life experiments…difficult to perform’” (ms. lines 110-11). Difficult indeed; practically impossible.

Against #6.

I document a clear pattern. Then, following a considerable body of earlier research that ms. lines 139-140 cite, I suggest a cause: higher support for research combined with Matthew effects that benefit those hired into PhD programs. The suggested causes correspond to the pattern documented. These are factors the nature of which Merton cited (and, as my text and I’m sure many others demonstrate, so have many subsequent scholars) as sources of particularism. Therefore, I believe that the result is both documented and significant, and suggest that it is consistent with particularism. To rescue his/her contrary logic, #6 cites no—none, not an iota of--supporting literature (although s/he might have cited the Coles’ highly tendentious studies) or evidence, and merely waves that magic refutation wand.

Mindful of ms. lines 107-124, I am entitled to turn #6’s logic—and #1’s, and yours—against all of you:

1. If hiring were truly meritorious, we should not see nearly the degree of overlap between program levels that is visible in career productivity measures. The abstract, which #6 parsed so laboriously on editorial grounds, clearly states this overlap. A pity that #6 didn’t also read it for its content.
2. If hiring were truly meritorious, we should see a strong correlation between bibliometric measures in precocity and career-long intervals that patterns in the data do not show.
3. Anyone who feels free to suppose freely to the contrary has certain commensurate obligations. One is to acknowledge, however begrudgingly, that his/her objections are speculative, not grounded. Another is to offer either evidence, not mere supposition, to the contrary, or at least to suggest positive, constructive ways independently to test at once one’s own suppositions and perhaps contrary views. It has been years, but I once read Popper. How, in the least way, is your contrary view falsifiable? If it is not, then how does it deserve serious regard, and what defense does any legitimate argument like mine have against it? Anyone can say “Yes, but….” *Prove* that I’m wrong, or at least offer grounded, perhaps even constructive, criticisms. Don’t instead freely *suppose* that I might be wrong, and thereby further suppose that you have refuted my argument or even so much as offered constructive criticisms. Free supposition is cheap. Put your proverbial money where your glaring presumptions are.

Beyond the ms.’s empirical and analytical contributions, lines 986-1013 and 1105-26 qualify results, suggest other kinds of evidence that might support or refute my tentative conclusion, and advocate research to collect and analyze such evidence. They demonstrate my willingness both to be proven wrong and to *constructively* suggest lines of research, not to freely suppose in armchair fashion.

Editors aren’t responsible for the quality of reviews, except so far as they take care to recruit informed, fair reviewers. But editors can—should—recognize when reviewers are unfair. Free supposition as a form of special pleading is plainly unfair, unreasonable, and invalid as criticism. A fair editor would discount it. You simply pass it on, with the requirement to revise in light of it. #1 and #6 made no grounded, substantive criticisms of study design, data, or analysis. #6 believed that s/he criticized the design, but only by recourse to undisciplined free supposition. I have not only disputed the criticism in reason, but presented empirical evidence against it. #1 and #6 don’t like the ms. Duh. Of course they’re entitled to their opinions. The ms. is entitled to substantive, grounded criticism, not the free supposition so freely exercised in their comments. “Yes, but…” “What if…?” “Maybe…” Free supposition is no basis for a fair professional review.

Sincerely,

Michael J. Shott
